# Increased Proportion of Dual-Positive Th2–Th17 Cells Promotes a More Severe Subtype of Asthma

**DOI:** 10.1155/2021/9999122

**Published:** 2021-08-05

**Authors:** Wenjin Sun, Yu Yuan, Lulu Qiu, Qingping Zeng, Jingsi Jia, Xudong Xiang, Aijun Jia, Libing Ma, Shaokun Liu, Bing Xiao

**Affiliations:** ^1^Department of General Medicine, West China Hospital, Sichuan University, Chengdu, Sichuan 610041, China; ^2^Lab of Lung Immuno-Inflammation, Frontiers Science Center for Disease-Related Molecular Network, Sichuan University, Chengdu, Sichuan 610041, China; ^3^Department of Pulmonary and Critical Care Medicine, The Second Xiangya Hospital of Central South University, Changsha, Hunan 410011, China; ^4^Department of Emergency, The Second Xiangya Hospital of Central South University, Emergency and Difficult Diseases Institute of Central South University, Changsha, Hunan 410011, China; ^5^Department of Respiratory Medicine, The Affiliated Hospital of Guilin Medical University, Guilin, Guangxi 541001, China; ^6^No. 3 Emergency Department of Yuelushan Hospital District, Hunan Provincial People's Hospital, Changsha 410000, Hunan, China

## Abstract

Asthma is a heterogeneous disease, and abnormal activation of T cells is the driving link of asthma's pathophysiological changes. Dual-positive Th2–Th17 cells, as newly discovered T-helper cells, have the functions of Th2 and Th17 cells and can coproduce Th2 and Th17 cytokines. Previous studies have shown that dual-positive Th2–Th17 cells increase the chances of asthma and correlate with asthma severity. However, the exact role of dual-positive Th2–Th17 cells in asthma is not known. Since there is no mature differentiation method for dual-positive Th2–Th17 cells, the present study aimed to clarify the strict differentiation conditions and reveal how dual-positive Th2–Th17 cells regulate asthma phenotypes. In this study, we confirmed that IL-1*β*, IL-6, anti-IFN-*γ*, and IL-21 promoted biphenotypic cell differentiation. Moreover, more proportion of dual-positive Th2–Th17 cells can be obtained by conditioned differentiation of mouse CD4^+^ T cells after classical allergic asthma modeling. Before asthma modeling, adoptive dual-positive Th2–Th17 cells promoted T cells to differentiate into the same biphenotype cells and exacerbated the severity of asthma. Together, our results clarify the differentiation conditions of dual-positive Th2–Th17 cells and further confirm that it stimulates asthma T cells to differentiate into the same biphenotype cells, leading to exacerbation of asthma.

## 1. Introduction

Bronchial asthma is a chronic inflammatory disease of the airways [1, 2]. Different subtypes can be seen in asthma patients due to their heterogeneity [3–5]. As an important part of the pathogenesis of asthma, the abnormal activation of T cells is the initiating factor leading to a series of pathophysiological changes [6, 7]. At present, a large number of studies have proved that T-helper cells have plastic deformability and transform each other under different stimuli [8–10]. The presence of dual-positive Th2–Th17 cells was first discovered in 2010 [11]. Further studies have shown that the proportion of dual-positive Th2–Th17 cells in asthmatic patients is significantly higher than that of healthy people [12]. As a newly discovered T-helper cell, however, no further study on dual-positive Th2–Th17 cells has been made.

Dual-positive Th2–Th17 cells have the functions of Th2 and Th17 cells and can secrete the characteristic cytokines of Th2 and Th17 simultaneously [11]. Some researchers have confirmed the existence of the Th2–Th17 cell population which can coexpress transcription factors GATA binding protein 3 (GATA3) and retinoic acid receptor-related orphan receptor gamma (ROR*γ*t) [13–16] and secrete both IL-4 and IL-17 in a mouse model of acute asthma [17]. This further proves that CD4^+^ T cells have the plasticity to transform into different T-cell subpopulations under appropriate environmental stimuli. But the differentiation conditions of dual-positive Th2–Th17 cells are not clear. It has also been clinically found that Th2/Th17^predominant^ asthma (dual-positive Th2–Th17 cells predominate in asthma) patients are more severe and have a poor response to inhaled corticosteroids [18–20]. The above studies show that the dual-positive Th2–Th17 cells may be an important cell subgroup in the occurrence and development of asthma. However, its mechanism in the pathogenesis of asthma is not very clear. Thus, elucidating the effect of dual-positive Th2–Th17 cells on the development and phenotype of asthma may provide new insight for the pathogenesis and prevention of asthma.

## 2. Materials and Methods

### 2.1. Mouse and Asthma Model

Female C57BL/6 mice were provided by the animal center of the Second Xiangya Hospital of Central South University (Changsha, China), weighing 18–20 g, aged 6-7 weeks. The asthma model was established as previously reported [21, 22]. Mice (*n* = 6/group) were given an intraperitoneal sensitization injection with 25 *μ*g Ovalbumin (OVA, Grade V, Sigma-Aldrich, USA) with 2 mg aluminum hydroxide (Sigma-Aldrich) on days 0 and 7. Then, mice were challenged with OVA solution atomized for 30 minutes on days 14, 15, 16, 17, 18, 19, and 20. Mice were sacrificed on day 21. All studies were performed in compliance with the Second Xiangya Hospital and Central South University Animal Care and Use Committee guidelines.

### 2.2. CD4^+^ T-Cell Differentiation

Spleen CD4^+^ T cells of mice were isolated by microbead sorting (130-049-201, Miltenyi Biotec, Germany). They were centrifuged and resuspended in 12-well flat-bottom plates, differentiated with 20 ng/mL of IL-1*β*, 20 ng/mL of IL-6, and 20 ng/mL of anti-IFN-*γ* in the presence of 10 ng/mL of IL-21 or 10 ng/mL of IL-23 [11, 12]. All cytokines were purchased from BioLegend Co. (BioLegend, USA). The cells were seeded in 12-well flat-bottom plates for 0, 1, 2, 4, and 6 days. Trypan blue dye exclusion staining was used for the cell viability analysis before cell culture, cytokines stimulus, flow cytometry, adoption, and other operations.

### 2.3. Flow Cytometry

After CD4^+^ T-cell differentiation, cells were stimulated with 3 *μ*g/mL of monensin, 1 *μ*g/mL of ionomycin, and 50 ng/mL of phorbol-12-myristate-13-acetate (PMA) for 5 h. Then, cells were fixed and permeabilized with fixation and permeabilization buffer for 15 min. Reagents used above were purchased from Multi Sciences Co. (Multi Sciences, China). After that, cells were washed with permeabilization buffer and then stained with intracellular markers PE-anti-IL-4 cytokine antibodies and APC-anti-IL-17 cytokine antibodies (BioLegend, USA) in the permeabilization buffer for 20 min. Cell surface marker FITC-anti-CD4 (Multi Sciences Company, China) cytokine antibodies were stained without fixed and permeabilized. Flow cytometry was conducted, and the data were analyzed using the FACSCalibur and FlowJo V10 software.

### 2.4. Enzyme-Linked Immunosorbent Assay (ELISA)

CD4^+^ T-cell culture supernatants after cytokine treatment and blood serum of mice after asthma modeling were collected for ELISA. IL-4 and IL-17 ELISA kits were used following the manufacturer's instructions (R&D, USA) to detect the cytokine secretion.

### 2.5. Quantitative Real-Time PCR

Trizol reagent (Invitrogen) was used for total RNA of CD4^+^ T cells isolation. The first-strand cDNA was synthesized with the RevertAid First Strand cDNA Synthesis Kit (Fermentas). Quantitative real-time PCR was performed using SYBRGreen Master Mix (ABI, Shanghai, China). Oligonucleotide primers for target genes were designed by Primer Premier 5.0 and synthesized by Sangon Biotech (Shanghai) Co., Ltd. China. The following primers were used: GATA3-forward, 5′-TCAGGGCACTAAGGGTTGTTAACTT-3′ and GATA3-reverse, 5′-GAATTCCATCCATGAGACACACAA-3′; ROR*γ*t-forward, 5′- CCTGGGCTCCTCGCCTGACC-3′ and ROR*γ*t-reverse, 5′-TCTCTCTGCCCTCAGCCTTGCC -3′; *β*-actin-forward, 5′- CATCCTGCGTCTGGACCTGG-3′ and *β*-actin-reverse, 5′-TAATGTCACGCACGATTTCC-3′. The mRNA expression values were normalized to *β*-actin. Each experiment was repeated 3 times in duplicates. The 2^−∆∆Ct^ method was used to analyze relative gene expression according to our pervious study [23].

### 2.6. Airway Hyper-Responsiveness (AHR) Assessment

On the 21^st^ day of the asthma model, methacholine- (Mch-) induced airway resistance was measured by direct plethysmography (Buxco Electronics, RC System, Wilmington, NC, USA) according to published methods [24, 25]. After tracheal intubation, we first measured mice baseline lung resistance for 1 min. Then, mice were given 10 *μ*L of atomized saline and 10 *μ*L of Mch at the following doses (0.39, 0.78, 1.56, and 3.12 mg/mL) to stimulate the airway and record RL for analyzing.

### 2.7. Bronchoalveolar Lavage Fluid (BALF) Processing

BALF was collected after 3 injections of 0.5 mL saline (37°C) into the lungs. The red blood cells were removed. The BALF cells after centrifugation (1500 rpm, 5 min, 4°C, Eppendorf centrifuge, Hamburg, Germany) were resuspended in phosphate buffer saline. BALF cells were counted using a hemocytometer. To obtain a BALF cell differential count, the cells were fixed and stained with Wright–Giemsa stain, and 200 cells were counted under a light microscope for statistical analysis.

### 2.8. Histopathology

The lungs were first slowly instilled in the trachea with 10% formalin, then removed, and stored in 10% formalin for fixation. The fixed lung tissue was embedded in paraffin and cut into thin slices (5 *μ*m) and then stained with hematoxylin and eosin (H&E). Stained sections were selected from each group and evaluated individually under a light microscope.

### 2.9. Statistics Analysis

All quantitative data were recorded as means ± SD. Data were analyzed using a *t*-test (two-group comparison) or one-way ANOVA (multigroup comparison). And, all the data were analyzed using SPSS software version 22.0. Differences between groups were considered statistically significant with a *p* value <0.05.

## 3. Results

### 3.1. Exploration of Dual-Positive Th2–Th17 Cells Differentiation Conditions

**Figure 1:**
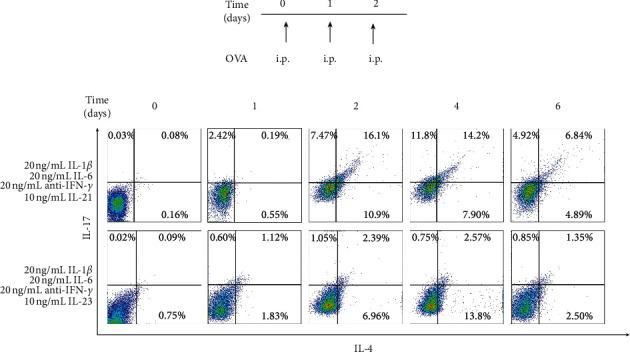
Cytokines IL-1*β*, IL-6, anti-IFN-*γ*, and IL-21 promote dual-positive Th2–Th17 cell differentiation. (a) Mice immunized intraperitoneally (i.p.) with 100 *μ*g of OVA for 3 days continuously. Then, spleen CD4^+^ T cells were isolated for cytokine interventions. 20 ng/mL IL-1*β*, 20 ng/mL IL-6, 20 ng/mL anti-IFN-*γ*, and 10 ng/mL IL-21 or 10 ng/mL IL-23 were added. After 0, 1, 2, 4, and 6 days of differentiation, cells were harvested for the following studies. (b) The proportion of dual-positive Th2–Th17 cells was assessed by flow cytometry. Data are the representative of at least three separate experiments.

So far, there is no mature differentiation method for dual-positive Th2–Th17 cells. The current controversy is whether IL-21 or IL-23 is the necessary cytokines for the differentiation of dual-positive Th2–Th17 cells, in the presence of cytokines IL-1*β*, IL-6, and anti-IFN-*γ* [11, 12]. We first explored the differentiation conditions. We performed the following experiments: CD4^+^ T cells were isolated 3 days after OVA intervention (Figure 1(a)), 20 ng/mL IL-1*β*, 20 ng/mL IL-6, 20 ng/mL anti-IFN-*γ*, and 10 ng/mL IL-21 or 10 ng/mL IL-23 were added for differentiation intervention, and flow cytometry was used to detect the effects of different intervention cytokines and intervention time on the differentiation of cells in each group. After 24 h, no significant differentiation trend was observed in the cells of each group (Figure 1(b)). At the 2^nd^, 4^th^, and 6^th^ days, we found a dramatical increased in dual-positive Th2–Th17 cells in the IL-1*β*/IL-6/anti-IFN-*γ*/IL-21 group, comparing with the IL-1*β*/IL-6/anti-IFN-*γ*/IL-23 group (Figure 1(b)). Among them, the best differentiation effect is at the 2^nd^ and 4^th^ days (Figure 1(b)). These data indicate that cytokines IL-1*β*/IL-6/anti-IFN-*γ*/IL-21 is the differentiation conditions for dual-positive Th2–Th17 cells. Particularly, IL-21 is necessary to promote the differentiation of dual-positive Th2–Th17 cells.

### 3.2. Increased Dual-Positive Th2–Th17 Cells after Differentiation Based on Asthma Modeling

**Figure 2:**
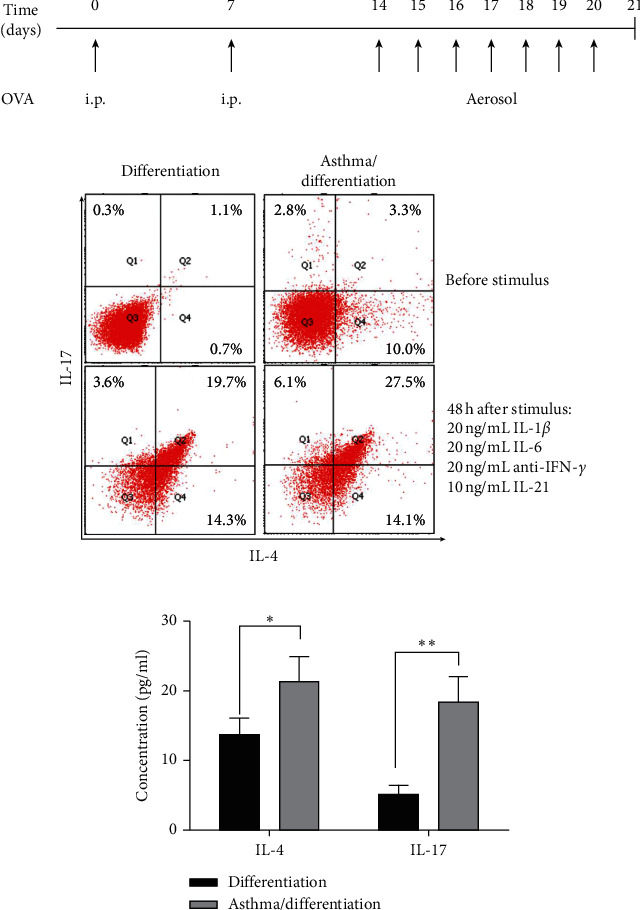
More dual-positive Th2–Th17 cells were obtained after conditional differentiation based on asthma modeling. (a) Mice received allergens to build the allergic asthma model (asthma/differentiation group) or saline separately (differentiation group). Twenty-four hours after last challenge, spleen CD4^+^ T cells were isolated for dual-positive Th2–Th17 cells conditional differentiation. Cells and supernatants were harvested for testing after 48 h of intervention. i.p. represents intraperitoneal injection. (b) The proportion of dual-positive Th2–Th17 cells was assessed by flow cytometry. (c) Secreted IL-4 and IL-17 in supernatants were evaluated using ELISA. ^*∗*^*p* < 0.05. ^*∗∗*^*p* < 0.01. Data are representative of at least three separate experiments.

Based on the differentiation conditions we have obtained, we next test whether differentiation based on asthma modeling can obtain more biphenotypic cells. C57BL/6 mice were used to build the asthma model (Figure 2(a)). Twenty-four hours after the last challenge, CD4^+^ T cells were isolated for differentiation. Forty-eight hours after cytokine stimuli, cells were collected for detection. In the asthma/differentiation group, there was an increased proportion of dual-positive Th2–Th17 cells between the differentiation group (Figure 2(b)). ELISA confirmed that IL-4/IL-17 was overexpressed in asthma/differentiation cells (Figure 2(c)). These results show that differentiation after asthma modeling stimulates more dual-positive Th2–Th17 cell differentiation.

### 3.3. Adoptive Dual-Positive Th2– Th17 Cells Promote T-Cell Differentiation in an Asthma Model

**Figure 3:**
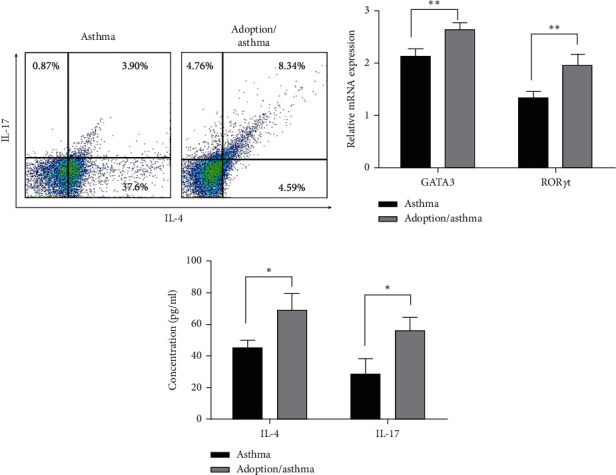
Dual-positive Th2–Th17 cells promote further differentiation and function of T cells into the same biphenotypic cells. Mice received dual-positive Th2–Th17 cells obtained from asthma/differentiation mice (adoption/asthma group) or naïve CD4^+^ T cells (asthma group) separately and then received allergens to build an asthma model. Twenty-four hours after the last challenge, spleen CD4^+^ T cells and blood serum were harvested for various studies. (a) The proportion of dual-positive Th2–Th17 cells was assessed by flow cytometry. (b) mRNA expression of GATA3 and ROR*γ*t in spleen CD4^+^ T cells was detected by quantitative real-time PCR. (c) Secreted IL-4 and IL-17 in supernatants were evaluated using ELISA. ^*∗*^*p* < 0.05. Data are the representative of at least three separate experiments.

The abnormal activation of T cells is the initiating factor leading to a series of pathophysiological changes in the pathogenesis of asthma. We hypothesized that dual-positive Th2–Th17 cells may alter T-cell differentiation and function in asthma. To this end, Th2–Th17 biphenotypic cells obtained from asthma/differentiation mice were transferred intravenously into C57BL/6 mice to build the asthma model. In adoption/asthma mice, we found that dual-positive Th2–Th17 cells proportion was shown to be increased compared to asthmatic mice (Figure 3(a)). Similarly, real-time PCR confirmed a substantial increase in the expression of key transcription factors GATA3 and ROR*γ*t in the adoption/asthma group (Figure 3(b)). Meanwhile, IL-4 and IL-17 show the same trend as GATA3 and ROR*γ*t (Figure 3(c)). Together, these data suggest that dual-positive Th2–Th17 cells promote further differentiation and function of T cells into the same biphenotypic cells.

### 3.4. Adoptive Dual-Positive Th2–Th17 Cells Cause More Severe Asthma Subtype in an Asthma Model

**Figure 4:**
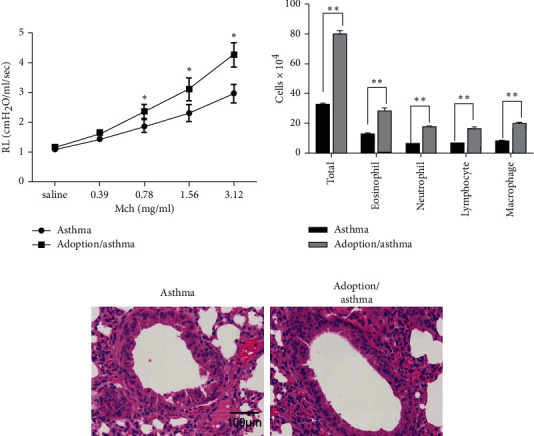
A more severe asthma subtype caused by dual-positive Th2–Th17 cell adoption. Mice received dual-positive Th2–Th17 cells obtained from asthma/differentiation mice (adoption/asthma group) or naïve CD4^+^ T cells (asthma group) separately and then received allergens to build an asthma model. Twenty-four hours after the last challenge, (a) lung resistance was assessed by a Buxco instrument in response to methacholine (Mch). (b) Total number, eosinophil, neutrophil, lymphocyte, and macrophage cells in bronchoalveolar lavage fluid. (c) Inflammatory cell infiltration in lung tissues was observed by H&E. Scale bar = 100 *μ*m. ^*∗*^*p* < 0.05. ^*∗∗*^*p* < 0.01. Data are representative of at least three separate experiments.

Clinically, patients with Th2/Th17^predominant^ asthma have severe symptoms and poor hormone treatment. We speculated that dual-positive Th2–Th17 cells may be involved in affecting the severity of asthma. After adoption and asthma modeling, mice were sacrificed for detection. Lung resistance and BALF cell count were measured as previously published [24, 25]. As shown in Figure 4(a), there was a significant increase in lung resistance in the adoption/asthma group compared with the asthma group. Also, BALF cell count suggested a significantly higher total number and as well as eosinophils and neutrophils number in adoption/asthma group compared with the asthma group (Figure 4(b)). Besides, compared with the asthma group, the pathological analysis showed that the adoption/asthma group had more inflammatory cell infiltration (Figure 4(c)). Taken together, these data indicate that dual-positive Th2–Th17 cells interventions could exacerbate AHR and airway inflammation, resulting in a more severe asthma subtype.

## 4. Discussion

As a newly discovered T-helper cell, dual-positive Th2–Th17 cells do participate in the occurrence and development of asthma [19, 20]. However, little research has been done on dual-positive Th2–Th17 cells. The reason is that there are no mature conditions for their differentiation. Theoretically, naïve CD4^+^ T cells, Th2 cells, or Th17 cells may have the potential to become dual-positive Th2–Th17 cells. Cosmi et al. confirmed that the differentiation of naïve CD4^+^ T cells into dual-positive Th2–Th17 cells in response to stimulation with IL-1*β*, IL-4 with IL-23 [11]. Notably, Th17 cells have the plasticity to become dual-positive Th2–Th17 cells in the presence of IL-4 [11]. While other researchers showed Th2-polarizing stimuli (IL-4) could induce Th17 cells to produce IL-4, but shut down their IL-17 production. Moreover, it was not IL-23, but IL-21 accompanied by IL-1*β*, IL-6, and anti-IFN-*γ*, which had the potential to produce dual-positive Th2–Th17 cells, especially when stimulating Th2 cells [12]. Study showed that IL-6 induced expression of IL-21 that amplified an autocrine loop to induce more IL-21 and IL-23 receptor in naïve CD4^+^ T cells. Both IL-21 and IL-23 can induce IL-17 expression [26]. At present, the controversy is mainly focused on whether IL-21 or IL-23 is the necessary cytokines for the differentiation of dual-positive Th2–Th17 cells. In our study, we tried to change the key cytokine to confirm that IL-21 can promote biphenotypic cell differentiation, and the optimal intervention time is 2–4 days. Not only this but we also found for the first time that more dual-positive Th2–Th17 cells can be obtained by conditioned differentiation of mouse CD4^+^ T cells after classical allergic asthma modeling. This laid a solid foundation for the subsequent research of dual-positive Th2–Th17 cells. Future studies are warranted to reveal the underlying mechanisms that drive the induction of dual-positive Th2–Th17 cells during allergic inflammation.

Dual-positive Th2–Th17 cells make up a very small portion among circulating CD4^+^ T cells in healthy subjects. Their numbers are increased in donors with asthma [11]. Other researchers have also found dual-positive Th2–Th17 cells in the peripheral blood of allergic asthma and further confirmed the existence of this cell subtype by constructing asthma models [27]. Subsequent studies on this type of cell mostly focused on the relationship between the characteristics of the cell and its proportion and the severity of asthma [19, 20]. Wang et al.'s experiment designed the effect of injection of dual-positive Th2–Th17 cells on asthma, but asthma modeling was received only 2 days, which was not enough to simulate asthma phenotype [12, 28, 29]. Beyond that, there are no studies on the use of dual-positive Th2–Th17 cells in the asthmatic model. For the first time, we constructed a classic allergic asthma model based on adoptive dual-positive Th2–Th17 cells and studied its effect on asthma subtypes. Our study found that dual-positive Th2–Th17 cells can not only promote further differentiation and differentiation of T cells into the same biphenotype cells but also exacerbate airway hyperresponsiveness and airway inflammation, leading to more severe asthma subtypes. These results suggest a significant role for dual-positive Th2–Th17 cells in asthma pathogenesis. Mechanism of this type of cell subsets regulating asthma subtype changes needs to be investigated further.

In conclusion, our data have clarified the differentiation conditions of dual-positive Th2–Th17 cells and further confirmed that it stimulates asthma T-cell differentiation and function, leading to exacerbation of asthma. This study could lead to new therapeutic prospects for the treatment of patients with more severe asthma.

## Data Availability

The data used to support the findings of this study are available from the corresponding author upon request.

## Abbreviations

AHR: Airway hyper-responsiveness
BALF: Bronchoalveolar lavage fluid
GATA3: GATA binding protein 3
OVA: Ovalbumin
ROR*γ*t: Orphan nuclear receptor.

## Supplementary Materials

Supplementary Materials Supplementary Figure 1: flow cytometry gating strategy. Supplementary Figure 2: cell viability analysis. (A) Trypan blue dye exclusion staining (×100) and (B) live cell proportion. Data are the representative of at least three separate experiments.

## References

[1] Martinez F. D., Vercelli D. (2013). Asthma. *The Lancet*.

[2] Reddel H. K., Bateman E. D., Becker A. (2015). A summary of the new GINA strategy: a roadmap to asthma control. *European Respiratory Journal*.

[3] Wenzel S. E. (2012). Asthma phenotypes: the evolution from clinical to molecular approaches. *Nature Medicine*.

[4] Zissler U. M., Esser-Von Bieren J., Jakwerth C. A., Chaker A. M., Schmidt-Weber C. B. (2016). Current and future biomarkers in allergic asthma. *Allergy*.

[5] Lloyd C. M., Hessel E. M. (2010). Functions of T cells in asthma: more than just TH2 cells. *Nature Reviews Immunology*.

[6] Moldaver D. M., Larché M., Rudulier C. D. (2017). An update on lymphocyte subtypes in asthma and airway disease. *Chest*.

[7] Sun W., Xiao B., Jia A. (2018). MBD2-mediated Th17 differentiation in severe asthma is associated with impaired SOCS3 expression. *Experimental Cell Research*.

[8] Mocci S., Coffman R. L. (1995). Induction of a Th2 population from a polarized leishmania-specific Th1 population by in vitro culture with IL-4. *Journal of Immunology*.

[9] Coffman R. L., Correa-Oliviera R., Mocci S. (1995). Reversal of polarized T helper 1 and T helper 2 cell populations in murine leishmaniasis. *Ciba Foundation Symposium*.

[10] Yang W., Chen X., Hu H. (2020). CD4+ T-cell differentiation in vitro. *Methods in Molecular Biology*.

[11] Cosmi L., Maggi L., Santarlasci V. (2010). Identification of a novel subset of human circulating memory CD4+ T cells that produce both IL-17A and IL-4. *Journal of Allergy and Clinical Immunology*.

[12] Wang Y.-H., Voo K. S., Liu B. (2010). A novel subset of CD4+ TH2 memory/effector cells that produce inflammatory IL-17 cytokine and promote the exacerbation of chronic allergic asthma. *Journal of Experimental Medicine*.

[13] Stark J. M., Tibbitt C. A., Coquet J. M. (2019). The metabolic requirements of Th2 cell differentiation. *Frontiers in Immunology*.

[14] Withers D. R., Hepworth M. R., Wang X. (2016). Transient inhibition of ROR-*γ*t therapeutically limits intestinal inflammation by reducing TH17 cells and preserving group 3 innate lymphoid cells. *Nature Medicine*.

[15] Zhang G., Zhang P., Liu H. (2017). Assessment of Th17/treg cells and Th cytokines in an improved immune thrombocytopenia mouse model. *Hematology*.

[16] Castro G., Liu X., Ngo K. (2017). RORgammat and RORalpha signature genes in human Th17 cells. *PLoS One*.

[17] Raymond M., Van V. Q., Wakahara K., Rubio M., Sarfati M. (2011). Lung dendritic cells induce TH17 cells that produce TH2 cytokines, express GATA-3, and promote airway inflammation. *Journal of Allergy and Clinical Immunology*.

[18] Cosmi L., Santarlasci V., Maggi L., Liotta F., Annunziato F. (2014). Th17 plasticity: pathophysiology and treatment of chronic inflammatory disorders. *Current Opinion in Pharmacology*.

[19] Irvin C., Zafar I., Good J. (2014). Increased frequency of dual-positive TH2/TH17 cells in bronchoalveolar lavage fluid characterizes a population of patients with severe asthma. *Journal of Allergy and Clinical Immunology*.

[20] Chiarella S. E., Budinger G. R. S., Mutlu G. M. (2015). *β*2-agonist therapy may contribute to the air pollution and IL-6-associated risk of developing severe asthma with dual-positive TH2/TH17 cells. *Journal of Allergy and Clinical Immunology*.

[21] Hoffman S. M., Tully J. E., Nolin J. D. (2013). Endoplasmic reticulum stress mediates house dust mite-induced airway epithelial apoptosis and fibrosis. *Respiratory Research*.

[22] Jia A., Wang Y., Sun W. (2017). Comparison of the roles of house dust mite allergens, ovalbumin and lipopolysaccharides in the sensitization of mice to establish a model of severe neutrophilic asthma. *Experimental and Therapeutic Medicine*.

[23] Yang H.-H., Duan J.-X., Liu S.-K. (2020). A COX-2/sEH dual inhibitor PTUPB alleviates lipopolysaccharide-induced acute lung injury in mice by inhibiting NLRP3 inflammasome activation. *Theranostics*.

[24] Locke N. R., Royce S. G., Wainewright J. S., Samuel C. S., Tang M. L. (2007). Comparison of airway remodeling in acute, subacute, and chronic models of allergic airways disease. *American Journal of Respiratory Cell and Molecular Biology*.

[25] Xu L., Sun W. J., Jia A. J. (2018). MBD2 regulates differentiation and function of Th17 cells in neutrophils-dominant asthma via HIF-1*α*. *Journal of Inflammation*.

[26] Zhou L., Ivanov I. I., Spolski R. (2007). IL-6 programs TH-17 cell differentiation by promoting sequential engagement of the IL-21 and IL-23 pathways. *Nature Immunology*.

[27] Liu W., Liu S., Verma M. (2017). Mechanism of TH2/TH17-predominant and neutrophilic TH2/TH17-low subtypes of asthma. *Journal of Allergy and Clinical Immunology*.

[28] Kianmeher M., Ghorani V., Boskabady M. H. (2016). Animal model of asthma, various methods and measured parameters: a methodological review. *Iranian Journal of Allergy, Asthma, and Immunology*.

[29] Aun M., Bonamichi-Santos R., Arantes-Costa F. M., Kalil J., Giavina-Bianchi P. (2017). Animal models of asthma: utility and limitations. *Journal of Asthma and Allergy*.

