# Supplementary figures and images for: Increased Proportion of Dual-Positive Th2–Th17 Cells Promotes a More Severe Subtype of Asthma
Source: Can Respir J. 2021 Aug 5;2021:9999122. doi: 10.1155/2021/9999122 (PMC8363460; doi:10.1155/2021/9999122)

## Slide 1
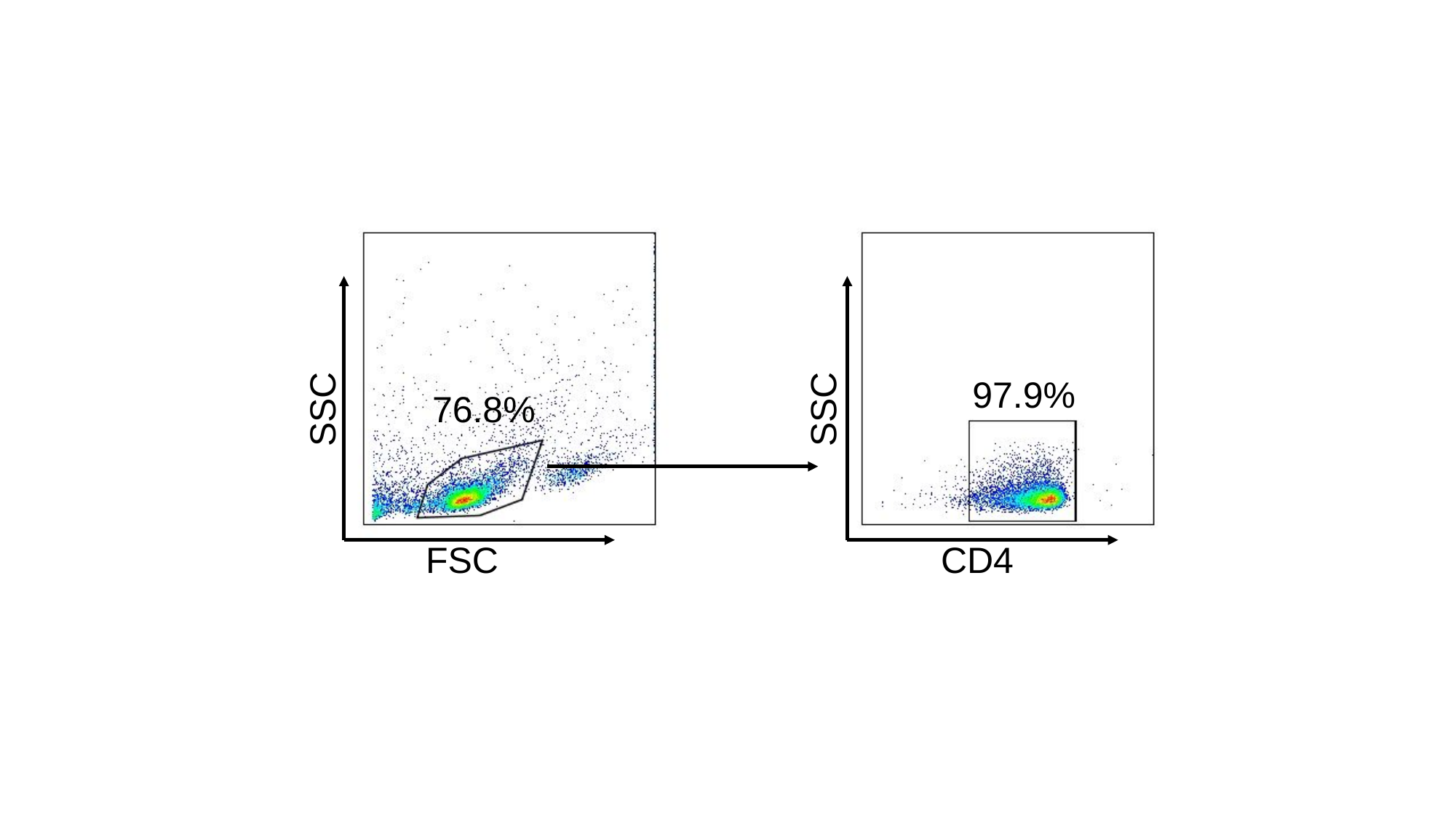

SSC
SSC
97.9%
76.8%
FSC
CD4

Supplement: Supplementary Materials — Supplementary Figure 1: flow cytometry gating strategy. Supplementary Figure 2: cell viability analysis. (A) Trypan blue dye exclusion staining (×100) and (B) live cell proportion. Data are the representative of at least three separate experiments. [file 9999122.f1.zip › supplementary figure 1 (2).pptx]

## Slide 1
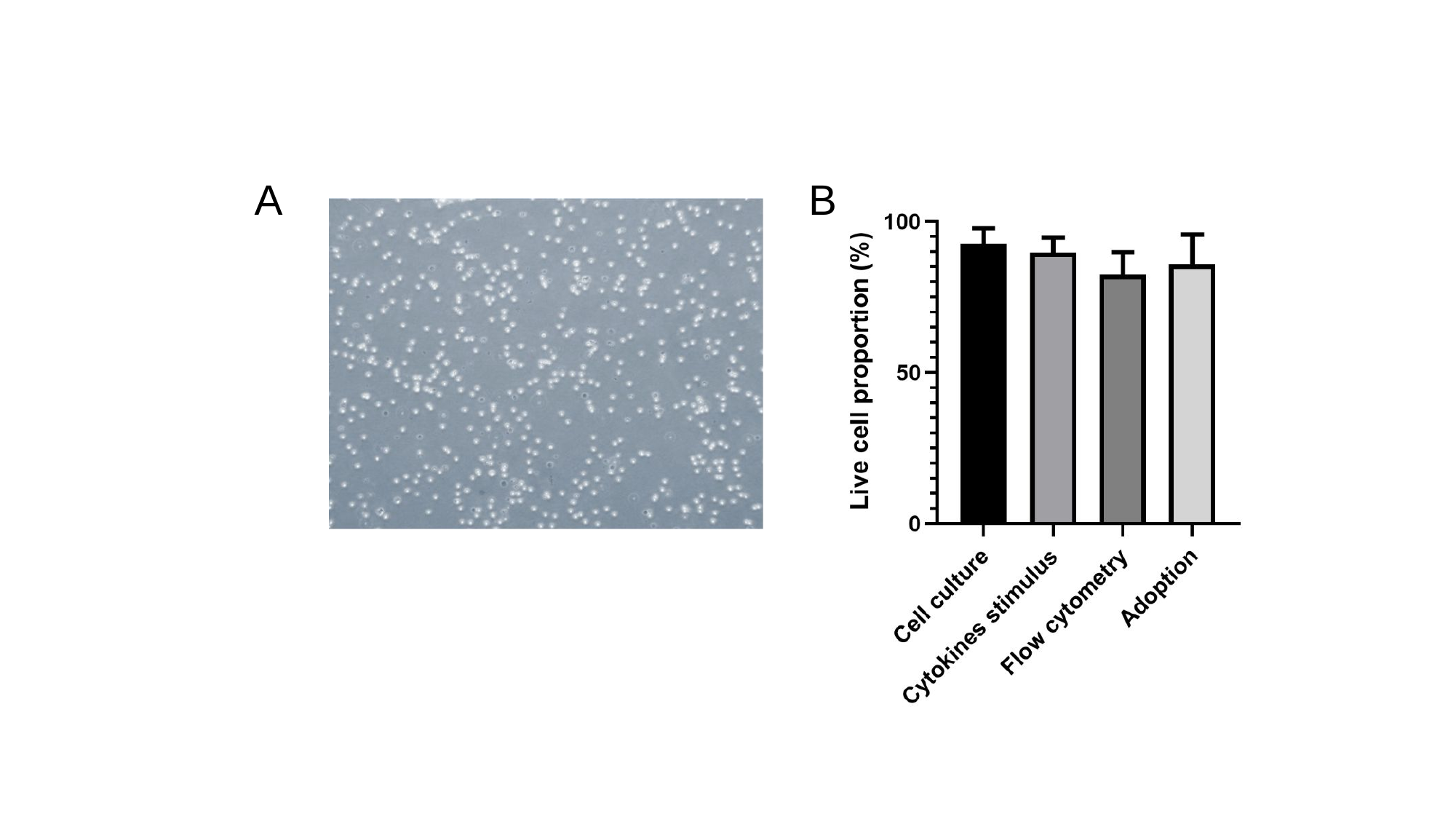

A
B

Supplement: Supplementary Materials — Supplementary Figure 1: flow cytometry gating strategy. Supplementary Figure 2: cell viability analysis. (A) Trypan blue dye exclusion staining (×100) and (B) live cell proportion. Data are the representative of at least three separate experiments. [file 9999122.f1.zip › supplementary figure 2 (3).pptx]
